# Data and Image Transfer Using Mobile Phones to Strengthen Microscopy-Based Diagnostic Services in Low and Middle Income Country Laboratories

**DOI:** 10.1371/journal.pone.0028348

**Published:** 2011-12-14

**Authors:** Coosje J. Tuijn, Bas J. Hoefman, Hajo van Beijma, Linda Oskam, Nicolas Chevrollier

**Affiliations:** 1 KIT (Royal Tropical Institute) Biomedical Research, Amsterdam, The Netherlands; 2 Text to Change, Kampala, Uganda; 3 Text to Change, Amsterdam, The Netherlands; 4 Netherlands Organization for Applied Scientific Research (TNO), Delft, The Netherlands; Mahidol Oxford Tropical Medicine Research Unit, Thailand

## Abstract

**Background:**

The emerging market of mobile phone technology and its use in the health sector is rapidly expanding and connecting even the most remote areas of world. Distributing diagnostic images over the mobile network for knowledge sharing, feedback or quality control is a logical innovation.

**Objective:**

To determine the feasibility of using mobile phones for capturing microscopy images and transferring these to a central database for assessment, feedback and educational purposes.

**Methods:**

A feasibility study was carried out in Uganda. Images of microscopy samples were taken using a prototype connector that could fix a variety of mobile phones to a microscope. An Information Technology (IT) platform was set up for data transfer from a mobile phone to a website, including feedback by text messaging to the end user.

**Results:**

Clear images were captured using mobile phone cameras of 2 megapixels (MP) up to 5MP. Images were sent by mobile Internet to a website where they were visualized and feedback could be provided to the sender by means of text message.

**Conclusion:**

The process of capturing microscopy images on mobile phones, relaying them to a central review website and feeding back to the sender is feasible and of potential benefit in resource poor settings. Even though the system needs further optimization, it became evident from discussions with stakeholders that there is a demand for this type of technology.

## Introduction

In many Low- and Middle Income-Countries (LMIC), access to health care is a challenge. Some of the bottlenecks are associated with long distances and poor communications between health care providers in (rural) health clinics and health centers at a higher level where better facilities and more experienced staff are often present. These limitations may lead to inequities in access to and quality of care [1]–[3].

Laboratory services are vital to public health, disease control and surveillance and individual patient diagnosis [4]. However, diagnosis is frequently based on clinical symptoms rather than on laboratory results [5]. Despite efforts to improve laboratory services, these continue to be one of the most neglected areas of health care provision all through LMIC. Poor and vulnerable populations are most affected by weak laboratory services because they carry the largest burden of ill health. Effective diagnosis of infectious diseases at both laboratory and community level will help reduce this burden [2], [6]. In many low resource settings, microscopy is one of the most important procedures for the diagnosis and control of various bacterial and parasitic infections, including tuberculosis, malaria and diarrhoeal, urinary and reproductive tract diseases [7]. Microscopy services are often suboptimal due to, for example, insufficient training and maintenance, poor slide preparation techniques, poor condition of the microscope, and low quality of essential laboratory supplies [8]. As a result, many common diseases are misdiagnosed and improperly treated, with far reaching consequences for individual patients and high costs for the health system [5], [8].

Modern means of communication, including mobile phones and the Internet, may be used to improve communication between the players in the health system. Increased availability of modern telecommunications has the potential to improve co-ordination within health systems. However reliable internet access remains poor in remote areas. Mobile phones are a notable exception since the coverage of mobile networks is already impressive and rapidly increasing even in remote areas. The number of mobile subscribers exceeds the number of fixed line connections in many LMIC [9]–[11].

Mobile phones offer an attractive possibility to address problems in accessibility, quality, effectiveness, efficiency and costs of health care. Several initiatives have shown previously that mobile phones can be used to take pictures through a microscope [8], [12]–[14] or be modified to serve as a microscope [15], [16]. The next logical step is to send the captured images via mobile networks, for example to a reference laboratory for confirmation of diagnosis by an expert.

For this study, we developed a system of mobile phone-based imaging in combination with a data transfer and response platform. We piloted the performance of this system in Uganda and discussed its acceptability and potential applications with local stakeholders.

## Methods

### Ethics Statement

Ethics approval or written informed consent was not required as this is a methodology proof of concept paper. Our focus was firstly on capturing a microscopy image of sufficient quality and then on the Information Technology (IT) part to send the captured image using the mobile phone. Patients' personal information or any samples ID's that might allow tracing of patients was not used.

Informed consent for discussions with colleagues at the health centers was obtained verbally prior to site visits.

### Location

In Uganda the health system is based on a referral system consisting of different levels, I indicating the lowest and IV the highest level; at higher levels more facilities and expertise are available [17]. The image capture was carried out by a laboratory technologist at the National Tuberculosis (TB) Reference Laboratory (NTRL) in Kampala district, two laboratory technicians, two assistants and a student at Nkozi Hospital laboratory in Nkozi, Mpigi district and five laboratory assistants at several level III and IV health centers from both these districts. The data transfer system was tested by a laboratory technologist and a doctor at the International Hospital of Kampala (IHK) and by IT specialists from a local IT company in Kampala.

### Materials used

Laboratory samples from the day before, or set aside for training purposes or external quality control of the health center were used in our study. These could not be linked to patients in any way.

Samples were initially collected as part of the routine activities of the laboratories and either made anonymous, or -in the case of blood slides for blood parasites- an unlabelled duplicate slide was sometimes used. In the case of urine we used a drop of urine that was placed on an unlabelled slide and put under the microscope for examination.

### Slide preparation

At NTRL and the TB health centres the slides selected for this study were sputum slides for TB training purposes or QC, samples were stained with Ziehl Neelsen. Blood slides for malaria that were used at Nkozi hospital and the health centres in the other sites were stained with Giemsa staining technique. Urine and stool slides were unstained direct wet preparations. Auramine staining was used for the detection of TB in sputum by fluorescent microscopy. Staining of samples was performed in a European laboratory.

### Image capture system

**Figure 1:**
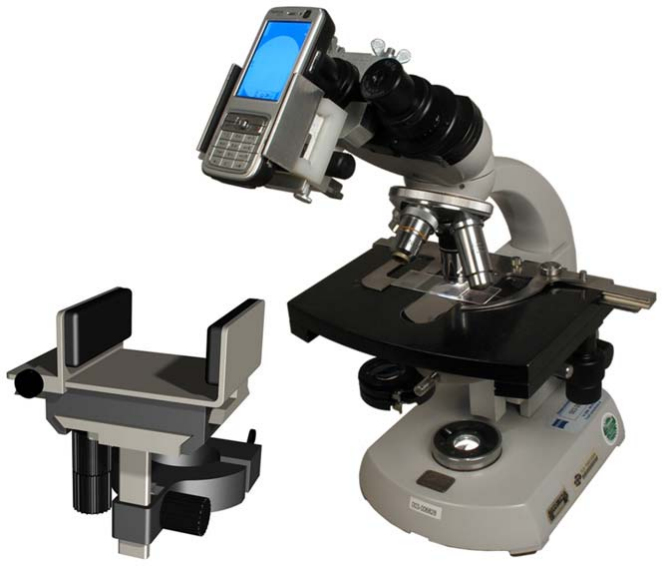
The prototype connector which attaches a mobile phone to a microscope. The prototype connector was designed by TU Delft and TNO. The connector is adjustable to fit all sizes of candy bar phones and some slide phones. It also consists of adjustable screws to fit most regular light microscopes.

The system is composed of a traditional light microscope connected via a connector used as a positioning device, to a Java-enabled mobile phone with camera, see Figure 1. The connector prototype was designed prior to this feasibility study [8]. For this study we used the 40× and the 100× oil immersion objectives of the Olympus CX 21, Olympus CX31, Olympus CX41 and Humascope (Human) light microscopes and the 40× objectives of the Olympus CX21 FluoLED, Olympus BX41 and Nikon AFX-IIA fluorescent microscopes. Different camera phones were used, mainly Nokia candy bar models, see Table 1 for an overview. The image resolution varied from 0.3 megapixels (MP) to 5MP. On some occasions the digital zoom of the mobile phone camera was used to enlarge a specific area of the microscopy field and magnify the micro-organism to be identified.

**Table 1:** Types of mobile phones and resolutions tested are shown.

Mobile phone brand and type (MP)	Camera display resolution (pixels)	Max image resolution (pixels)	Comments
Sony Ericsson K320i (0.3)	128×160	640×480	Not sufficient quality for interpretation of pathogen
Nokia (1.3)	240×320	1280×960	Not sufficient quality for interpretation of pathogen
Nokia 6300 (2)	240×320	1600×1200	See Fig. 2, panel A. With a 100× oil immersion objective 2MP is sufficient
Samsung E900 (2)	240×320	1600×1200	Similar quality to that of Nokia 6300 (Data not shown)
iphone 3 (2)	320×480	1600×1200	Display quality was sufficient for interpretation, not used extensively (Data not shown)
Nokia N73 (3.2)	240×320	2048×1536	See Fig. 2, panel B. With a 100× oil immersion objective 2MP or more is sufficient
Nokia N82 (5)	240×320	2592×1944	Same as for iphone (data not shown).
Sony Ericsson k850i (5)	240×320	2592×1944	Display quality was sufficient for interpretation, see Fig. 2, panel C.
Nokia N95 (5)	240×320	2592×1944	Display quality was sufficient for interpretation, similar to the 5MP image in Fig. 2, panel C. (Data not shown)
Nokia C-3 (5)	240×320	2592×1944	See Fig. 3. With a 40× objective 5MP is required

### Data transfer system

End user and server software of the image transfer system were designed. A draft form for use on the mobile phone was developed for the end user to record ID, sample type, health center data, and upload images from the mobile phone.

An end user version of this software, including mobile Internet, was pre-installed as an extra application on a Java enabled mobile phone. A server version of this image transfer system was installed locally in Kampala, Uganda. We used Orange as our mobile internet provider as they cover both Mpigi and Kampala districts [18]. The end user recorded fictional information on the draft form, took the required picture(s), and pressed “send”. The image was then sent automatically via the pre-installed mobile Internet connection of the mobile phone, stored in the database of the central server and a confirmation text message that the image had been received was sent to the end user. Anyone with access to the central server could log on via an Internet page to this server and examine the received image, store it elsewhere, or give instant feedback via a text message or voice call, to the end user's phone. Additionally, the image could be enlarged on the computer screen using the digital zoom function of the computer in order to magnify areas of interest.

### Testing procedures

#### 1 a. Capture of image without connector

Laboratory staff members at Nkozi hospital, NTRL and the health centres were asked to place a sample slide under the microscope and focus. A short demonstration was given to show how to capture an image through the eyepiece. Then they were asked to use their camera phone to capture an image of the focused specimen through the eye piece. If they did not have a phone with camera, they could borrow one of the study phones.

#### 1 b. Image capture with connector

Next, the same laboratory staff members were given the connector and after a 10–15 minute demonstration, asked to attach the connector and a mobile phone and take a picture of what they saw through the microscope. In some cases assistance was given to fix the prototype connector on to the microscope and again a short demonstration was given on how to capture an image. Pictures were analyzed by the head of the laboratory or an experienced technician to determine whether parasites or bacteria were clearly visible and recognizable.

**2. At IHK we tested the data transfer system, by investigating the feasibility of the following steps:**

- Sending images and forms from the mobile phone to the website/platform.
- Log on and access the image at the central platform.
- Use of digital zoom within web application to magnify relevant part of image if indicated.
- Fill in the reply box and reply to the end user by means of a text message (feedback).

Medical and laboratory staff at IHK together with staff from the local IT company that developed the system tested the web interface.

### Group discussions

At Nkozi hospital we interviewed all four laboratory staff as a group on the user friendliness and expected demand. We also interviewed three laboratory staff as a group at the HC. Due to limited availability, individual questions were asked to some of the laboratory assistants in remote centers and to students as we thought they may bring up other issues. Other stakeholders in the health care chain (national programme representatives, Ministry of Health delegates), were asked for input on how the system could be used. Discussions were carried out using open ended questions.

## Results

### Imaging

Urban and rural hospital laboratories and health centers of levels III and IV were visited in Kampala and Mpigi district in Uganda. Laboratory staff (technologists, technicians and assistants) quickly understood the steps needed to capture a clear microscopy image. No prior training was given other than a short demonstration of how to capture an image through the eyepiece using the camera phone. This was done with and without using a connector. A connector was not essential for capturing microscopy images, however using one made it much easier and stabilized the phone enabling clear image capture. In some cases support was needed to position the connector correctly and place the mobile phone. It became evident that the mobile phone connector did not fit all oculars of every microscope type securely. This was the case with the Humascope and some of the Olympus CX series where the bottom of the ocular had a thicker rubber or metal ring and the screws of the connector were not long enough to fasten the connector tightly, resulting in friction or slight movement of the phone during positioning of the camera. Nevertheless, we showed that the principle works.

**Figure 2:**
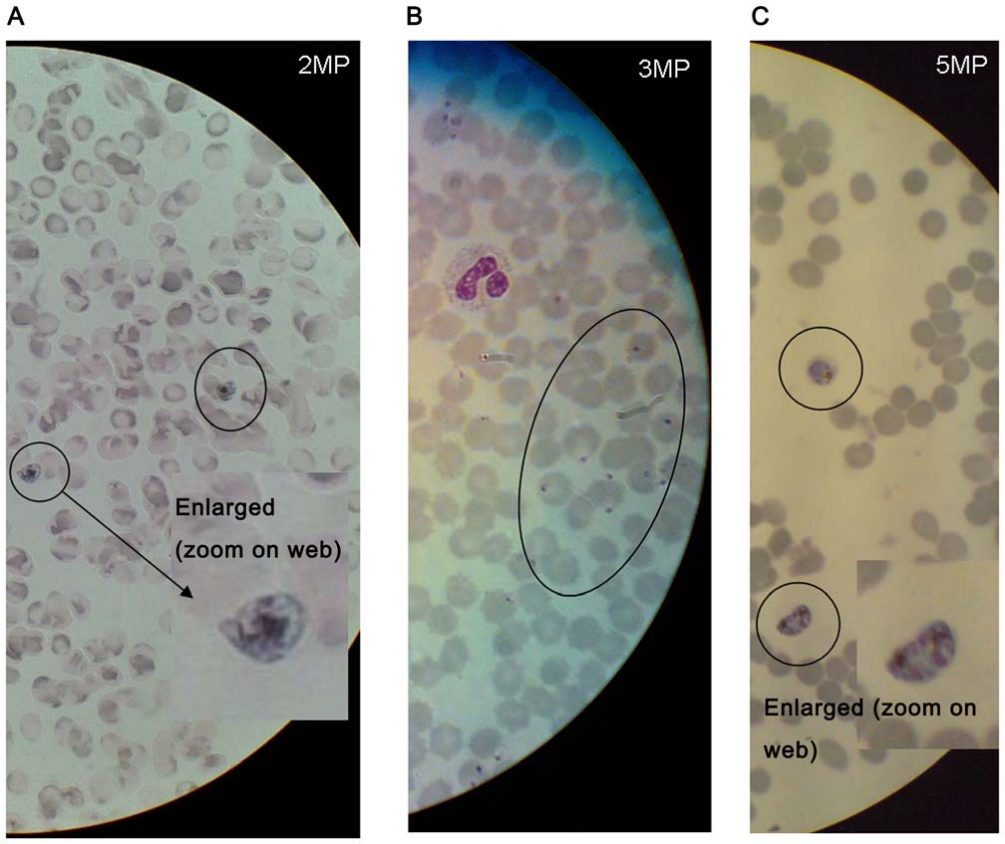
Pictures from malaria parasites stained with a regular Giemsa stain and visualized by light microscopy (field 100× oil immersion) taken with mobile phone cameras with different resolutions. Panel 2A shows a 2 MP image and panel 2C a 5MP image of malaria parasites. Inside the black circles different stages of the parasites (schizonts and gametocytes) are visible. The middle panel 2B shows a 3MP image of a white blood cell and inside the black circle, malaria trophozoites.

**Figure 3:**
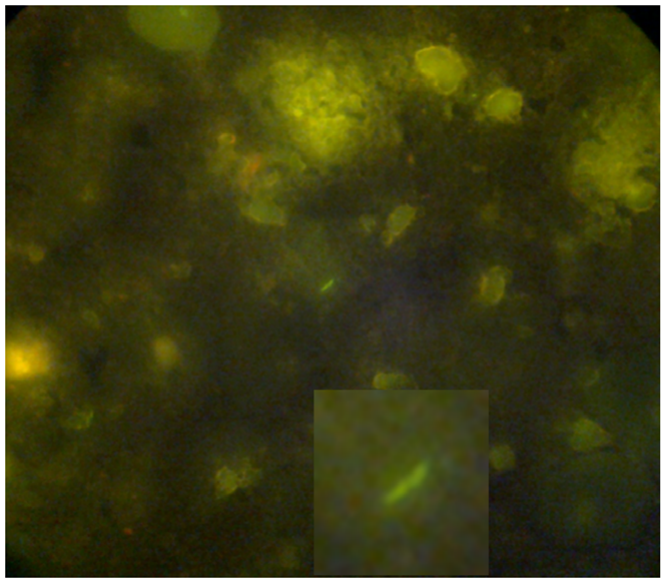
A 5MP image of an auramine stained sputum sample captured in our European laboratory. The image was captured with a Nokia C3 mobile phone on a Nikon AFX-IIA microscope (field 40×). *Mycobacterium tuberculosis* is depicted in the center and a magnification of the bacterium is shown at the bottom of the image.

Figure 2 shows several images of a malaria parasite taken by Nokia and Sony Ericsson phones with 2, 3, and 5 megapixel cameras. We found that using a high magnification on the microscope (100× oil immersion) required at least a 2MP camera to obtain a clear image, see table 2. Malaria parasites were so clearly visible on the mobile phone screen that it was often possible to identify the parasite stage (see Figure 2). To obtain a clear image of a TB bacterium stained with Ziehl Neelsen proved to be more difficult as the TB bacteria are small, 1–4 microns in length. If bacteria were not abundant it was challenging to capture a good image suitable for diagnosis or confirmation. Fluorescent microscopy provided a better result, shown in figure 3 and as fluorescent microscopy is nowadays recommended for TB diagnosis [19] it was important to include this in our study. Several images of auramine stained TB bacilli were captured, but due to the suboptimal position of the connector on this particular microscope (Olympus FluoLED CX 21) we were unable to obtain a clear image in Uganda. Fluorescent microscopy was performed using the 40× objective and required a 5MP camera for obtaining clear images (table 2). One facility we visited only had a microscope with mirror as there was no electricity available, but we could still use the phone camera to take a picture of a microscopy image (results not shown).

**Table 2:** Microscopy diagnostic applications and mobile phone camera resolutions tested.

Pathogens of disease	Size of microbe	Staining technique	Resolution tested	Microscope objective	Result
Tuberculosis (*Mycobacterium tuberculosis*)	1–4 µm	a. Ziehl Neelsenb. Fluorescent stain kit	a. 2MP or 5MPb. 5MP	a. 100× oilb. 40×	a. Interpretable for verification of positive casesb. Not interpretable in Uganda due to connection problem, but interpretable in Europe (Fig. 3)
Malaria (*Plasmodium* parasites)	1–15 µm	Giemsa	2MP, 3MP or 5MP	100× oil	Interpretable for verification, species identification and diagnosis
Bacterial vaginosis (multiple bacteria)	±3 µm	Gram stain	2MP or 5MP	100× oil	Interpretable for verification, some species identification and diagnosis
Common Stool parasites, eggs (pathogenic)	>6 µm	No stain used, direct wet preparation	short video 30fps5MP	40×40×	Interpretable via parasite movement capture by film (i.e *Giardia lamblia* trophozoite).

We found that by using the video option of the mobile phone, it was possible to capture images from direct sample preparations of stool and urine containing live, mobile microbes. In this way morphology and specific movement of urine and stool parasites can be observed. Table 1 and 2 summarize our findings.

### Data transfer

**Figure 4:**
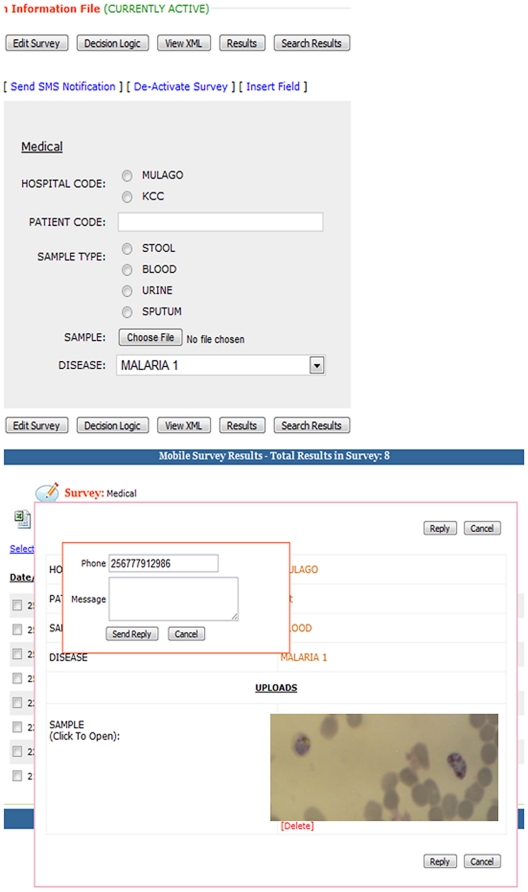
Data transfer system forms. Illustrated above is the test form on the end user's mobile phone. The image below shows the website page model for receiving the data (image, sample type and locations specifications). Images can be enlarged on the site and comments to image sent back to the requester through a text message.

The draft form, a tailor made messaging platform constructed for the end user to record ID, sample type, health center data and upload images on the mobile phone is shown in Figure 4 (above).

The form was filled in, an image was added, and the completed form was sent to the server using 3 G mobile internet. The sizes of the images sent varied between 70 kb–350 kb. Within minutes, the form could be accessed on a computer connected to the server via the Internet, Figure 4 (below). We were able to enlarge the image or specific parts of the image and reply by means of a text message.

### MMS

Several attempts were made in Kampala to send pictures from a mobile phone using Multimedia Messaging Service (MMS) as an alternative for mobile Internet. We found that the images were received late or never arrived. In addition, MMS was expensive and time consuming.

### Applicability and feasibility

We discussed with various stakeholders (see methods section) their ideas about the use of mobile phone imaging for improving diagnostic services. This provided insight in where this system could be most beneficial. The four main recommended activities for this system were: (i) confirmation of diagnosis/results; (ii) knowledge sharing/continuous education; (iii) quality control; and (iv) communication of results between laboratory and clinical staff at one location.

Staff explained that the larger hospital laboratories would have a computer and more experienced staff that could analyse their submitted images for confirmation or quality control purposes and send back a response by text message. They also pointed out that it would be useful if images were sent from one rural health center to another with the aim to share knowledge amongst staff of peripheral laboratories. Furthermore we suggested to the interviewees that a database could be created containing field examples to learn and share data; this idea was popular amongst students.

## Discussion

Long distances and poor communication result in inadequate access to health care in many LMICs [1]–[3]. Here we combine microscopy with mobile phones and mobile Internet to show that image capture combined with data transfer could be realized in Uganda. The sites visited in Uganda are largely representative of others in East Africa. The aim was to determine the feasibility of using mobile phones for capturing microscopy images and transferring these to a central database for evaluation, feedback and educational purposes. The future objective is to improve diagnosis in peripheral health settings by empowering undereducated and insufficiently experienced health care and laboratory workers to meet quality standards. The long term goal is to improve diagnosis in order to reduce mortality and morbidity.

Image capture was simple once the connector and mobile phone were in place and the staff was enthusiastic to see the microscopy image on their phones. The main bottleneck was to mount the connector to the microscope; the connector will need adjustment to become more user-friendly. In our experiments numerous people showed that it was not easy to capture a clear image by hand. They found that the camera was difficult to stabilize, centre and focus. End users were very responsive to using a connector as using hands only would require numerous trainings. However it is possible to capture an image without the use of a connector.

Generally, microscopy fields seen using naked eye examination are of higher quality than images captured by mobile phone, though for analysis of malaria parasites and with a phone camera resolution of 2MP or more, images were often so clear that specific stages of the malaria parasite could be identified. This is useful for treatment strategies. Microorganisms in urine and stool, which are sometimes morphologically difficult to determine were captured using short video, of which the file size was slightly larger than that of an image. Additional to the microbes mentioned in table 2, we assume that short video can also be used for verification of other common pathogenic stool (i.e. *Ascaris lumbricoides*) and urine parasite stages, (*i.e. Trichomonas vaginalis*, Schistosoma *haematobium*) as well as for identification of trypanosomes in chancre or cerebrospinal fluid.

A remaining challenge is TB diagnosis, which often requires the examination of many microscopic fields. Here, mobile phone imaging could be used for confirmation of positive cases. Alternatively we showed that it is possible to use a mobile phone in combination with fluorescence microscopy as a lower magnification (40×) is required and thus fewer microscopic fields need to be scanned. The smaller the magnification of the microscope the more camera pixels necessary for a good image and the best results with fluorescent microscopy were obtained with a camera resolution of 5MP. The use of these images for quality control purposes, which was one of the recommended activities of this system, is still limited due to the lower resolution compared to naked eye examination.

In a future study it is important to consider the preparation (staining) of samples, microscope settings and other factors that may influence the quality of the images. Additionally, a formal blinded assessment of diagnostic performance should be included for instance with slides containing different malaria species.

Image capture of microscopy images has been tried before with and without use of connecting devices or modifications to the mobile phone [12]–[16], [20]. The novel aspect of the current study is the combination of mobile phone imaging with data transfer via mobile Internet and feedback. Transferring data from the mobile phone to a website/platform can be done by Bluetooth, MMS or mobile Internet:

- Bluetooth can only work in combination with a computer close by; the maximum distance for Bluetooth is 10 meters.
- In contrast to earlier published data [12], [13] we found that sending an image by MMS in remote areas was not possible and even in Kampala remained slow, unreliable, and very expensive as the costs are always per image.
- It was easy and quick to send images from the mobile phone to a central server using the mobile Internet which is paid for per month independent of the amount of data sent. One limitation of the system is that currently only one or a few images (appr. 250 kb) can be attached to one message. However, for most applications this will not be a problem.

For providing feedback, the steps to log on to the server, access the sent image, zoom in when necessary, and send back a reply from the central platform to the mobile phone were straightforward. If zooming is required then something to consider is that the larger the image on a computer screen, the greater the amount of pixels that are needed. In addition, using the mobile phone's digital zoomed images for data transfer is not recommended as this would strongly limit the resolution of the image.

This pilot study used a bottom-up approach. End users and stakeholders were included in the design process and the initial testing of the system, increasing the chance of our concept to meaningfully improve laboratory services and support health workers and practitioners in their diagnosis and training. The possibility of constructing a connector locally, thereby reducing costs and enhancing local sustainability was introduced and discussed, yet further investigation and development is required and will be carried out in the next phase.

This study also included discussions on applicability and feasibility with laboratory/health care workers and other stakeholders in the health care chain. Laboratory staff emphasized the importance of sharing knowledge, receiving feedback and being able to learn to identify diseases that are rare or not (yet) present in their region. Together, we identified possibilities for improvement of quality control, better monitoring of disease, improved microscopy performance and improving confidence by being able to get in touch with other staff and experts. Most people thought that this system could facilitate the improvement of diagnosis by reducing misdiagnosis and incorrect treatment. Also it would benefit students who could gain access to a database of real life samples, rather than studying perfect text-book illustrations.

We observed that in Kampala most people in the laboratory have phones with cameras but not always with pre-installed Java software and a mobile Internet subscription, which are both essential for our system. This is also the case for health care workers in rural/remote areas. During discussions it was suggested that this could be overcome by supplying a camera phone to laboratories through programme funds or through the government system (with phone restrictions to avoid excessive and inappropriate use). Furthermore it was mentioned that the costs for mobile Internet, currently around 30 euro per month [21], could be incorporated in a service package.

Connecting mobile technology to diagnosis has a considerable potential to improve diagnostic services in resource poor settings with widely distributed and remote clinical centres. This field study proves that the concept works and that there is a demand from the end user and great interest from other stakeholders. It sets the stage for the next phase, during which several connector prototypes and various formats of the mobile forms need to be designed and the whole system tested in a controlled setting with one reference hospital supervising a limited number of peripheral clinics.

## References

[1] Eckman B, Gerdtham UG (2006). Macroeconomics and Health in Uganda, Mission report to the WHO.. http://www.who.int/macrohealth/action/CMH_UGA_Oct06_final.pdf.

[2] Mosha JF, Conteh L, Tediosi F, Gesase S, Bruce J (2010). Cost implications of improving malaria diagnosis: findings from north-eastern Tanzania.. PLoS One.

[3] O'Donnell O (2007). Access to health care in developing countries: breaking down demand side barriers.. Cad Saude Publica.

[4] Nsubuga P, Nwanyanwu O, Nkengasong JN, Mukanga D, Trostle M (2010). Strengthening public health surveillance and response using the health systems strengthening agenda in developing countries.. BMC Public Health.

[5] Petti CA, Polage CR, Quinn TC, Ronald AR, Sande MA (2006). Laboratory medicine in Africa: a barrier to effective health care.. Clin Infect Dis.

[6] Sekhri N (2006). From Funding to Action: Strengthening Healthcare Systems in Sub-Saharan Africa.. https://members.weforum.org/pdf/whitepaper.pdf.

[7] Wongsrichanalai C, Barcus MJ, Muth S, Sutamihardja A, Wernsdorfer WH (2007). A review of malaria diagnostic tools: microscopy and rapid diagnostic test (RDT).. Am J Trop Med Hyg.

[8] v Limmeren R, Chevrollier N, Esser PE, Kandachar P (2009). Microscope and Mobile Phones: Product Development in Uganda.. http://mhealthinfo.org.

[9] Asangansi I, Braa K (2010). The emergence of mobile-supported national health information systems in developing countries.. Stud Health Technol Inform.

[10] Krohn R (2010). There's an app for that–mHealth takes center stage.. J Healthc Inf Manag.

[11] Teltscher S, Gray V, van Welsum D, Biggs P, Magpantay E (2010). World Telecommunication/ICT Development Report 2010.. http://www.itu.int/dms_pub/itu-d/opb/ind/D-IND-WTDR-2010-PDF-E.pdf.

[12] Bellina L, Missoni E (2009). Mobile cell-phones (M-phones) in telemicroscopy: increasing connectivity of isolated laboratories.. Diagn Pathol.

[13] Frean J (2007). Microscopic images transmitted by mobile cameraphone.. Trans R Soc Trop Med Hyg.

[14] Godse CS, Patkar S, Nabar NS, Amonkar AJ, Vaidya RA (2008). Mobile Camera Microphotography: A Simple But Elegant Technique For Telediagnosis of Marlaria.. Journal of Medical Education and Research.

[15] Tseng D, Mudanyali O, Oztoprak C, Isikman SO, Sencan I (2010). Lensfree microscopy on a cellphone.. Lab Chip.

[16] Breslauer DN, Maamari RN, Switz NA, Lam WA, Fletcher DA (2009). Mobile phone based clinical microscopy for global health applications.. PLoS One.

[17] Ministry of Health Uganda..

[18] Orange Coverage website.. http://www.orange.ug/mobile-plans/coverage.php.

[19] Steingart KR, Henry M, Ng V, Hopewell PC, Ramsay A (2006). Fluorescence versus conventional sputum smear microscopy for tuberculosis: a systematic review.. Lancet Infect Dis.

[20] Zimic M, Coronel J, Gilman RH, Luna CG, Curioso WH (2009). Can the power of mobile phones be used to improve tuberculosis diagnosis in developing countries?. Trans R Soc Trop Med Hyg.

[21] Uganda Communications Comission (2010). Post and Telecommunications Market Review.. http://issuu.com/weinformers/docs/uganda_communication_statistics.

